# Robust technological readings identify integrated structures typical of the Levallois concept in Guanyindong Cave, south China

**DOI:** 10.1093/nsr/nwz192

**Published:** 2019-11-21

**Authors:** Yue Hu, Ben Marwick, Jia-Fu Zhang, Xue Rui, Ya-Mei Hou, Wen-Rong Chen, Wei-Wen Huang, Bo Li

**Affiliations:** 1 Department of Archaeology, Sichuan University, China; 2 Centre for Archaeological Science, School of Earth and Environmental Sciences, University of Wollongong, Australia; 3 Department of Anthropology, University of Washington, USA; 4 MOE Laboratory for Earth Surface Processes, Department of Geography, College of Urban and Environmental Sciences, Peking University, China; 5 Key Laboratory of Vertebrate Evolution and Human Origins, Institute of Vertebrate Paleontology and Paleoanthropology, Chinese Academy of Sciences, China; 6 CAS Centre for Excellence in Life and Paleo-environment, China; 7 Qianxi Bureau of Cultural Relics Protection, China; 8 ARC Centre of Excellence for Australian Biodiversity and Heritage, University of Wollongong, Australia

## INTRODUCTION

Recent technological readings of stone artefacts from Guanyindong Cave site, south-west China, have identified the Levallois strategy in sedimentary deposits dated to approximately 170 000–80 000 years ago. Some specimens in the assemblage are susceptible to misinterpretation. Here, we provide further detailed descriptions of these specimens to clarify that they possess attributes consistent with the Levallois concept, as it has been widely employed elsewhere.

We welcome the contribution by Li *et al.* [1] to the debate about the Levallois concept found in Guanyindong. Detailed discussion of terminology, definitions and assemblage descriptions plays an important role in clarifying new claims about the human past. We are pleased that Li *et al.* [1] have created an opportunity for further discussion to improve our understanding of the Middle Palaeolithic complex in south-west China. In our reply, we identify the key disagreements that Li *et al.* [1] have with our claims and respond with further details to clarify our position.

### LEVALLOIS CONCEPT

**Figure 1.**
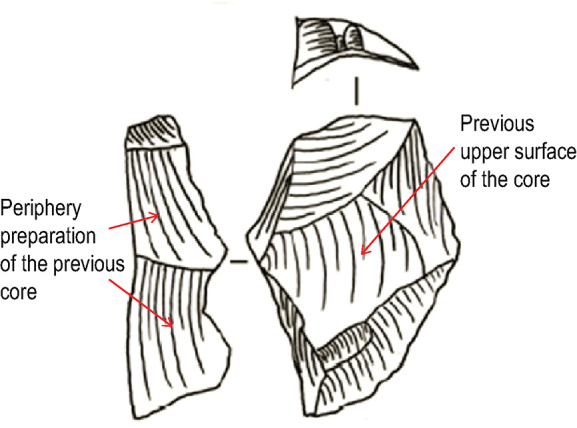
Illustration of débordant.

Li *et al.* [1] claim that we have misused the Levallois concept and presented a technological misreading of the Guanyindong lithic artefacts. We understand that a variety of non-Levallois methods of production can produce a few Levallois-like products in nearly any lithic assemblage. That is why we use the ‘volumetric method’ to avoid the possibility of misidentification. However, Li *et al.* [1] claim that we use an ‘*anachronistic typological approach*’, which we assume they mean a classification system based on a essentialist assumptions of real, discontinuous and immutable ‘kinds’ of unordered monothetic classes. This is incorrect. Our narrative in the Supplementary Information (SI) of Hu *et al.* [2] described our materialist, polythetic approach to classification that is orthogonal to a typological approach. In brief, to define a Levallois assemblage, we need to find a whole toolkit including cores, flakes and by-products, among which cores are the most crucial factor. We strictly followed the most widely used method, developed by Boëda. During our inspection of the artefacts, we confirmed their hierarchical structure, traces of preparations before knapping, the angle of percussion and the relationship between the flaking plane and plane of intersection of the two surfaces. For Levallois flakes, which are less directly demonstrative of the Levallois concept than cores, we inspected their dorsal scar pattern, the percussion angle, the relative ratio between length and width, platform size and preparation. Following these inspections, we found there are 31 flakes that were likely made according to the Levallois strategy. For by-products, which are mainly débordants, we see the original peripheral preparation of the core left on one side of the dorsal surface (Fig. 1). Those previous parts of the flaking platform indicate the maintenance of core convexity. To claim that we only used one or two criteria to define Levallois technology is an unfortunate misreading of our original publication.

Our narrative about specimen P15948 clearly describes our identification of the Levallois strategy in terms of hierarchically related sequences of flake removals. Our narrative emphasizes the process of shaping the piece to maintain the distal and literal convexities and organize these sequences. As we note in that text, this is typical of our approach to analysing these artefacts, but space does not permit presentation of a narrative for every artefact depicted in the illustrations in the paper.

There are many possible core morphologies that are consistent with the six technological criteria currently used to identify Levallois concepts [3–5]. These variations of morphologies may result in a wide range of shapes, but this does not alter the fundamental model of Levallois reduction. Our use of the Levallois concept is therefore well within the range of previously accepted uses in the scholarly literature.

### LEVALLOIS CORES

Li *et al.* [1] claim that many simple cores on flakes yield large, flat preferential flakes but meet none of the other criteria defining Levallois. Our analyses go beyond the basic identification of hierarchical relationships and preferential removals. In our SI, we illustrated our approach at length using core P15948 (Hu *et al.* 2019 Extended Data Fig. 5a) as an example of one core that meets Levallois criteria.

**Figure 2.**
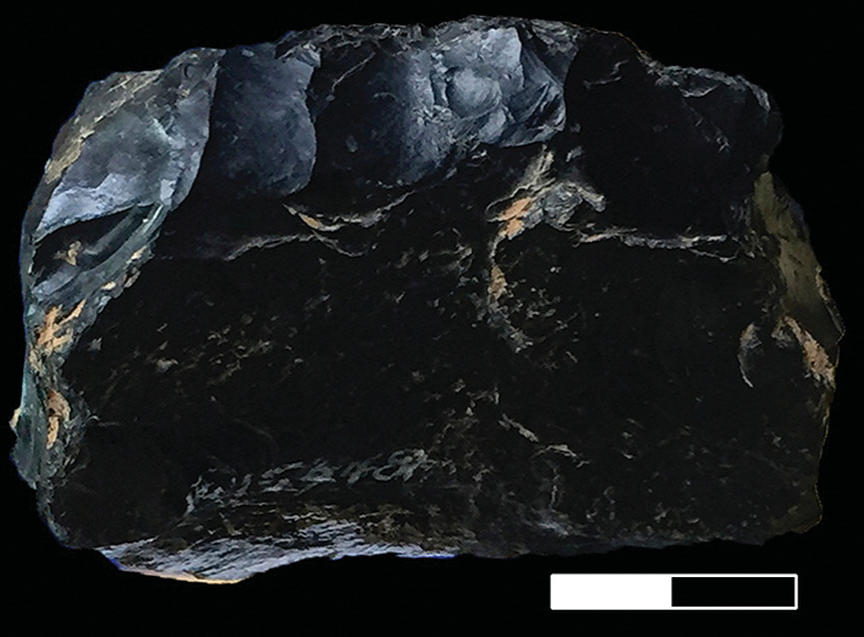
Details of platform preparation of P15948. Divisions on scale are 1 cm.

The Guanyindong assemblage contains few text-book-ideal Levallois cores. For example, we recognize that specimen P15948 may be mistaken as lacking platform preparation and having been worn by geological processes. We present here in Fig. 2 a detailed view of this specimen showing that platform has, in fact, been prepared. This is evident from the small, parallel-oriented, overlapping, step- and feather-terminated flake scars visible at the top of the image. Those faceting scars are the result of preparation, rather than taphonomic processes. We consider these to be preparation scars because the directions of the scars are all from the edge of periphery to the lower surface of the core and the scars have similar sizes and shapes. If they were caused by natural processes, then we would expect the scars to be oriented in multiple directions, to be located on multiple edges of the artefact and to have a wider range of sizes and shapes, reflecting a wide dynamic range of percussion energies.

A further misinterpretation that may occur with P15948 is that it may be mistaken as a flake where the lower surface of the flake has natural convexity to make determined products. However, it lacks unambiguous criteria that are widely recognized for identification of a specimen as a flake. It is a nodule with a similar thickness from end to end, with no visible traces that can be used to identify proximal or distal ends, or orient the specimen along a flaking percussion axis. There are no landmarks that can ascribe this nodule as the lower surfaces of a flake.

### ARTEFACTS WITH NATURAL ASYMMETRICAL SURFACES AND NATURAL CONVEXITIES

Li *et al.* [1] reject the possibility of artefacts with naturally asymmetrical surfaces being compatible with identification of Levallois technology and state they are unaware that this has been previously used in the identification of Levallois technology. Levallois cores we identified in our publication were carefully shaped as the example of Levallois core P15948 shows above. However, even if natural asymmetric surfaces were used, that detail cannot disqualify them as Levallois cores. Several studies identify cores with a morphology of naturally asymmetric surfaces as we do, even when the cores lack extensive flake removals to shape the core in preparation for the main flake removals [6–10]. Similarly, Brantingham and Kuhn [3] state: ‘Thus, pieces of raw material that begin with appropriate natural convexities and require little additional preparation could fall within the volumetric Levallois definition alongside heavily prepared centripetal cores that meet the strict typological definition.’ Pieces with naturally asymmetric surfaces within the Levallois concept are safely within the range of typical uses of this concept in the scholarly literature.

### READING OF ARTEFACT P4265

**Figure 3.**
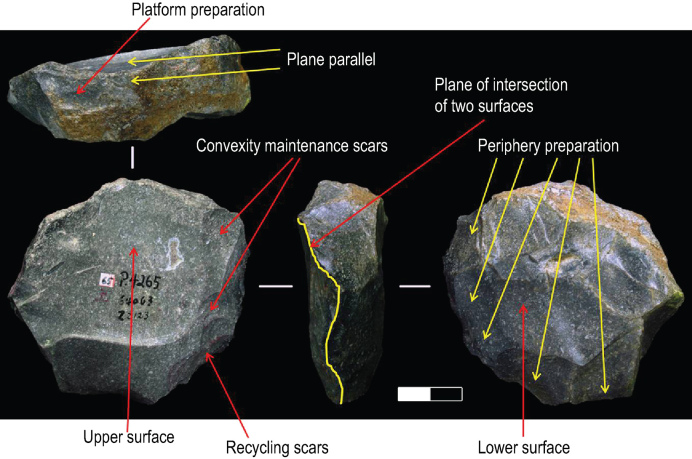
Illustration of Levallois core P4265. Divisions on scale are 1 cm.

Li *et al.* [1] present an alternative reading of artefact P4265, claiming that some of the small scars were produced after the detachment of the large flake, and so were unrelated to shaping the Levallois surface. The small scars on P4265 do not disqualify the core from identification as a Levallois core. We have annotated a photograph of this piece in Fig. 3 to illustrate our technological reading and further describe it below.

First, the core is hierarchically organized with the upper and lower surfaces intersecting a plane. The plane is parallel with the fracture plane. Second, the scars on the right side of the preferential scar can be divided into two parts: the first parts is a set of convexity preparation scars that were not entirely removed by the preferential scar; the second part of those scars represents two events. The first event was the production of flakes before the removal of the preferential scar, to help create the convexity. However, the natural convexity may have already been sufficient to make the predetermined products, so they did not overlap the preferential scar. The second event was the production of flakes after the preferential flake removal, which we can call ‘recycling scars’. These recycling scars may be aimed at preparing the second series of percussion, as defined in Boëda's method for preferential cores, where every successful production would be followed by re-preparation [11].

### IDENTIFICATION OF LEVALLOIS FLAKES

Li *et al.* [1] dispute our identification of some flakes as Levallois. Regarding specimen i in Fig. 3 and specimen i in Extended Data Fig. 6 of Hu *et al.* [2], Li *et al.* [1] claim this cannot be Levallois because no platform preparation is evident. However, the platform of a Levallois flake does not have to be prepared on every specimen. Numerous Levallois flakes from Europe and Levant have been described with a plain platform. For example, Soriano and Villa [12] describe a Levallois flake from central Italy in their Fig. 4B that has an unprepared platform. Soriano and Villa noted that this flake has a natural surface and further describe that this flake could fit in the Levallois strategy because its orientation and location of the natural surface on the platform was suitable for flaking. Similarly, Foltyn and Kozlowski [13] described 41 Levallois flakes from Poland; 20 of these have unprepared platforms and two have cortical platforms. Platform preparation is thus not a compulsory attribute for identification of the Levallois strategy on flakes.

On specimen i in Fig. 3 in Hu *et al.* [2], we see very slight waves that can indicate the direction of the applied force. It is unmistakably not a natural fracture, as claimed by Li *et al.* [1] We are confident of this because, in the Guanyindong assemblage, natural factures can be identified due to slight weathering resulting in yellow or brown surface discoloration, which we cannot find on this scar.

In the case of the Levallois flake depicted in Hu *et al.* [2] Extended Data Fig. 6g, we dispute the claim by Li *et al.* [1] that the point of impact for this flake is on the right corner. We find the point of impact in the central region of the platform and so the critique of Li *et al.* [1] on this issue can be dismissed because it fails to demonstrate how possible misidentification of some attributes would change the identification of the specimens from Levallois to non-Levallois.

### EXPLOITED PORTION OF CORES

Li *et al.* [1] claim that the exploited and unexploited portions of the cores have no apparent association. The ‘exploited’ and ‘unexploited’ portions of all the Levallois cores at Guanyindong form an integrated structure. One step cannot be achieved without the previous steps, as we have previously narrated in detail in our SI. Those steps include the creating lower surface and upper surfaces, making them hierarchically structured and preparing the striking platform for convexity to maintain flake production. The cores served as a whole to produce the final, predetermined products. We cannot see that they can be regarded as ‘additive’.

In sum, the Guanyindong Levallois is within the range of variation of descriptions of Levallois assemblages found elsewhere in the world. We are confident that, in a blind test, where the site of the artefacts was not known to the specialist who was presented with these pieces, these pieces would be indisputably identified as Levallois. They are unlikely to be mistaken for natural and opportunistic. We agree that unexpected, novel findings such as the Levallois at Guanyindong should be subject to high levels of scientific scrutiny. However, the new findings not fitting with one's prior expectations does not justify the application of a narrower definition for identifying the Levallois than has been used in other regions.
